# Evolutionary Insights into Irisin/FNDC5: Roles in Aging and Disease from *Drosophila* to Mammals

**DOI:** 10.3390/biom15020261

**Published:** 2025-02-11

**Authors:** Kiwon Lee, Myungjin Kim

**Affiliations:** Department of Molecular & Integrative Physiology, University of Michigan, Ann Arbor, MI 48109, USA; kiwonlee@umich.edu

**Keywords:** Irisin, FNDC5, exercise, aging, Iditarod, *Drosophila*

## Abstract

The Irisin/FNDC5 protein family has emerged as a pivotal link between exercise and the prevention of age-associated diseases. Irisin is highly expressed during exercise from skeletal and cardiac muscle cells, playing a critical role in mediating systemic health benefits through its actions on various tissues. However, Irisin levels decline with age, correlating with a heightened incidence of diseases such as muscle weakness, cardiovascular disorders, and neurodegeneration. Notably, the administration of Irisin has shown significant potential in both preventing and treating these conditions. Recently, an Irisin/FNDC5 homolog was identified in an invertebrate *Drosophila* model, providing valuable insights into its conserved role in exercise physiology. Importantly, Irisin/FNDC5 has been demonstrated to regulate autophagy—a process essential for clearing excessive nutrients, toxic aggregates, and dysfunctional organelles—in both flies and mammals. Dysregulated autophagy is often implicated in age-related diseases, highlighting its relevance to Irisin/FNDC5’s functions. These findings deepen our understanding of Irisin/FNDC5’s roles and its potential as a therapeutic target for mitigating aging-related health decline. Further studies are needed to elucidate the precise mechanisms by which Irisin regulates autophagy and its broader impact on physiological aging and related diseases.

## 1. Introduction

Aging is an irreversible biological process characterized by the progressive deterioration of physiological function across all body systems, leading to age-associated diseases such as neurodegenerative diseases [1,2], cardiovascular diseases [2,3], diabetes [2], musculoskeletal diseases [2], and even cancer [1,2,3]. With advancements in medical technology and improvements in living conditions, human life expectancy has significantly increased [4]. Consequently, understanding the biological mechanisms underlying the aging process is crucial for developing effective treatments and preventive strategies for age-related diseases, ultimately enhancing quality of life. Various biological processes and molecular mechanisms that change during aging—such as genomic instability [5], accumulation of nuclear DNA damage [2], alterations in DNA methylation [6], changes in nuclear architecture [7], telomere shortening [8], and mitochondrial dysfunction [8]—are being actively investigated to deepen our understanding of aging and its associated diseases.

**Figure 1:**
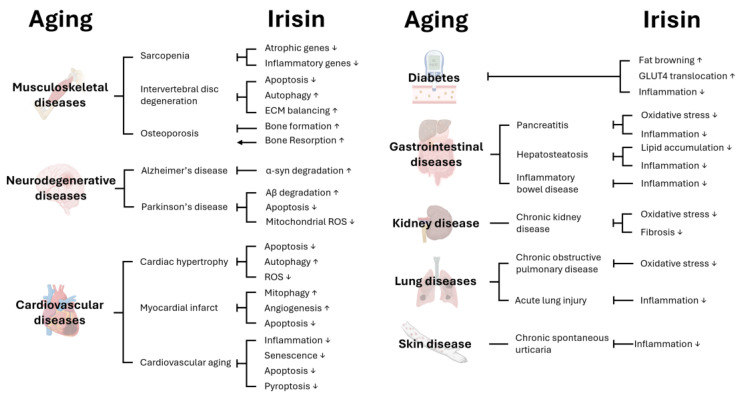
Effects of Irisin on age-associated diseases. Various biological processes are regulated by Irisin; therefore, Irisin can affect aging as well as age-associated disease in different tissues. This figure was created with BioRender (http://www.biorender.com).

Fibronectin type III domain-containing protein 5 (FNDC5) is prominently expressed in muscle [9]. Upon cleavage by the ADAM protease family at the plasma membrane, FNDC5 is secreted into the bloodstream, and it influences various tissues [10,11,12]. Serum Irisin level and *FNDC5* expression level are upregulated after exercise, which is known as physical training for preventing age-associated diseases [13,14,15]. Furthermore, Irisin has been linked to numerous age-associated diseases, including cardiovascular diseases [16], musculoskeletal diseases [17,18], neurodegenerative diseases [19], and diabetes [20]. Notably, *FNDC5* expression decreases with aging [21], and the *FNDC5* gene was shown to be critical for exercise benefits in attenuating various age-associated pathologies (Figure 1), including cardiovascular diseases [22]. Thus, Irisin appears to be closely associated with aging and age-related diseases. While many excellent reviews have highlighted the role of Irisin in various physiological systems [23,24,25,26], this review uniquely emphasizes recent findings that connect mammalian research with insights from *Drosophila* genetic studies. These studies, leveraging the evolutionary conservation of Irisin proteins, offer a deeper understanding of its mechanisms in autophagy, mitochondrial health, and tissue integrity. By integrating these findings with the existing literature, we aim to provide a comprehensive framework that highlights the translational potential of model organism research for addressing age-related diseases.

## 2. FNDC5/Irisin and Its Role in Autophagy

### 2.1. FNDC5/Irisin in Mammals

**Figure 2:**
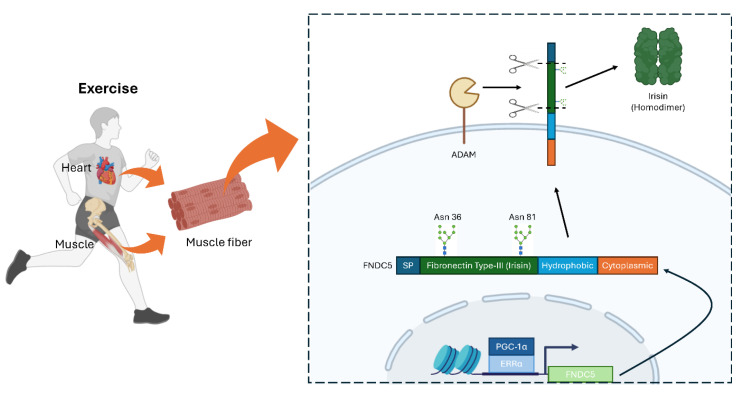
Expression and secretion of mammalian FNDC5/Irisin in muscle and heart. Exercise induces FNDC5 expression in PGC-1α/ERRα-dependent manner. After expression, FNDC5 undergoes cleavage on fibronectin type-III domain at the plasma membrane by ADAM protease. Irisin (cleaved FNDC5) forms a homodimer and circulates through blood vessels. SP, signal peptide. This figure was created with BioRender (http://www.biorender.com).

FNDC5 is a protein primarily expressed in skeletal muscle during physical exercise (Figure 2). Notably, the FNDC5 gene in humans contains an atypical ATA start codon rather than the canonical ATG found in other primates (chimpanzees, gibbons, gorillas) and most mammals (mice, rats) [27]. Although ATA is often considered a null mutation, this variant still generates a functional FNDC5 protein in humans [28]. The expression of FNDC5 is regulated by the transcription coactivator PGC-1α and the transcription factor ERRα, both of which are upregulated by exercise in skeletal muscle [13,29]. Exercise can induce PGC-1α and ERRα expression in skeletal muscle, subsequently leading to an increase in Irisin levels [30]. Epigenetic mechanisms also modulate FNDC5 expression, with histone H3 acetylation enhancing and H3 K27 di-methylation reducing FNDC5 levels [31].

Different exercise modalities and intensities distinctly influence the secretion of Irisin. In a randomized crossover trial on youth (12–18 years old), high-intensity interval training (HIIT) led to higher Irisin levels compared to moderate continuous intensity (MCI) exercise in those at a healthy weight, whereas no significant changes were observed in participants who were overweight or obese [32]. A systematic review and meta-analysis similarly reported that HIIT produced greater increases in serum Irisin than other exercise protocols among overweight or obese adults, with stronger effects in younger, overweight individuals versus older or obese populations [33]. Resistance training has likewise been shown to elevate circulating Irisin levels, particularly in older adults. In aging mice and humans, resistance exercise significantly boosted both serum and muscle Irisin expression, accompanied by enhanced grip and leg strength, suggesting a role for Irisin in preventing age-related decline in muscle function [34]. Taken together, these findings underscore the critical influence of exercise type, intensity, and individual factors—such as weight status and age—on Irisin dynamics.

Structurally, FNDC5 comprises four main regions: a signal peptide, a fibronectin type-III domain, a hydrophobic transmembrane domain, and a cytoplasmic domain [35]. After FNDC5 is inserted into the plasma membrane, members of the ADAM protease family cleave off the fibronectin type-III region, releasing the secreted peptide known as Irisin [10]. N-glycosylation at Asn-36 and Asn-81 is crucial for the stability and secretion of Irisin [36]. Once released, Irisin forms homodimers and acts on multiple tissues, including the heart, brain, bone, and adipose tissue, mediating diverse physiological effects [29,37,38,39,40].

Recent work has identified several Irisin-binding partners. Integrin αVβ5, found in osteocytes and adipose tissue, mediates Irisin-induced changes in bone turnover and adipocyte metabolism [41,42]. TGF-β receptor II in osteoblasts and kidney cells has also been reported to bind Irisin, suggesting potential roles in bone homeostasis and renal function [43,44]. Moreover, Irisin can be internalized via extracellular vesicles [45,46], and evidence suggests it may bind mitochondrial SOD2 in cardiomyocytes [47], implying a link to intracellular antioxidant defenses. Overall, these findings highlight the multifaceted roles of FNDC5 and Irisin in exercise physiology and age-related disease.

### 2.2. FNDC5/Irisin Regulate Autophagy in Mammals

Autophagy is an important lysosomal degradation pathway, which is critical for removing excessive nutrients, toxic aggregates, and damaged organelles such as oxidative stress-producing dysfunctional mitochondria [48]. In cells, the autophagy-initiating protein kinase ULK1 is important for autophagy control [49]. During nutrient affluence, mTOR kinase phosphorylates ULK1 and, therefore, autophagy is inhibited [50]. However, upon diverse stresses, such as nutrient, energetic, oxidative, hypoxic, and proteostatic stresses, ULK1 is activated to upregulate autophagic activities [50,51,52]. Protein kinases, such as AMPK can phosphorylate and modulate ULK1 activity in these stress contexts. In addition, autophagy induction could also be mediated through a ULK1-independent pathway.

Irisin has recently been shown to modulate autophagy [53]. Under insulin-resistant conditions, Irisin promotes the nuclear translocation of key autophagic regulators, such as TFEB, thereby increasing LC3II levels and decreasing p62, ultimately enhancing autophagic flux [54]. In hepatocytes, Irisin deficiency exacerbates steatosis and impairs autophagy via reduced AMPK phosphorylation and enhanced mTOR signaling, whereas Irisin supplementation reverses these effects, preventing lipid accumulation and restoring fatty acid oxidation [55]. Similar benefits extend to cardiomyocytes, where Irisin alleviates ischemia- and pressure-overload-induced damage by stimulating AMPK/ULK1-driven autophagy, preserving mitochondrial integrity, and reducing oxidative stress [56,57,58]. Moreover, Irisin-induced autophagy has been implicated in the survival of pancreatic β-cells in hyperglycemic conditions by activating AMPK/SIRT1/PGC-1α signaling, reducing apoptosis, and improving insulin secretion [59]. Taken together, these findings suggest that Irisin-induced autophagy provides a mechanistic link between exercise and its wide-ranging metabolic and cytoprotective effects across multiple tissues [53].

### 2.3. Isolation of Drosophila FNDC5/Irisin Homolog as a Novel Autophagy Mediator

Although Irisin was initially characterized in mammals and primarily linked to exercise-related metabolic benefits, it was unclear whether a functional equivalent existed outside the vertebrate lineage. The recent identification of *Iditarod* (abbreviated as *Idit*) in *Drosophila* melanogaster addressed this gap by revealing that fruit flies possess a homolog of the mammalian FNDC5/Irisin precursor protein [60]. Idit was first uncovered in a genetic screen aimed at identifying autophagy regulators downstream of the pro-autophagy kinases Atg1 and Atg13. In the screening, silencing *Idit* markedly suppressed excessive autophagy, suggesting an evolutionarily conserved pro-autophagic role akin to mammalian Irisin/FNDC5, similar to what was described above. Further domain mapping, phylogenetic analyses, and structure predictions confirmed that Idit shares a single Irisin-like fibronectin III domain and transmembrane region with vertebrate FNDC5, underscoring a deep evolutionary conservation of this protein family across the animal kingdom [60].

Structurally, *Drosophila* Irisin (Idit) mirrors key features of mammalian FNDC5: it contains an N-terminal signal sequence, a central Irisin domain composed predominantly of beta-sheet motifs, a predicted cleavage region, and a hydrophobic transmembrane region. AlphaFold and RoseTTAFold modeling indicate that the Irisin domain of Idit is highly similar to the crystallized human Irisin structure, including a likely glycosylation site and the capacity to form a dimer interface [60]. The Idit intracellular tail also appears slightly homologous to the corresponding region of mammalian FNDC5; however, *Drosophila* Idit’s intracellular domain is noticeably longer than that of mammalian FNDC5, suggesting that it could harbor additional intracellular interaction sites and regulatory motifs. This discrepancy in tail length raises the possibility that, in flies, Idit may contribute more extensively to intracellular signaling or membrane-trafficking events, beyond the primarily secretory and extracellular functions highlighted in mammalian Irisin/FNDC5.

### 2.4. Drosophila FNDC5/Irisin Homolog Controls Autophagy in a Cell-Autonomous Manner

Indeed, in *Drosophila*, Idit operates as a key downstream effector of Atg1/Atg13-dependent autophagy signaling [60], a role that diverges somewhat from mammalian Irisin/FNDC5, which is best known for circulating myokine and endocrine effects and may function upstream of the ULK1 complex, mammalian Atg1/Atg13 counterpart [55]. *Idit* silencing through RNAi suppresses Atg1-Atg13-induced autophagy and autophagic cell death, while the overexpression of *Idit* triggers ectopic autophagy in diverse tissues, including fat bodies and muscle [60]. Unlike mammalian systems—where secreted Irisin also elicits systemic metabolic changes—*Drosophila* Idit seems to exert a more cell-autonomous regulation of autophagosome formation. Since the mechanistic studies of how Irisin controls autophagy were not thoroughly conducted in different contexts of mammalian physiology, it is possible that Irisin/FNDC5 in mammals may have cell-autonomous and ULK1 downstream functions similar to *Drosophila* Idit. These findings also suggest that Irisin/FNDC5 homologs can function through both local intracellular cues and long-range signals to promote autophagy in a context- and species-dependent manner.

**Figure 3:**
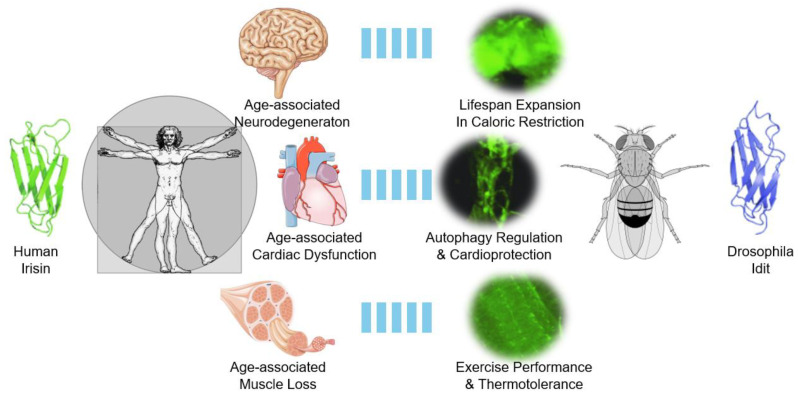
*Drosophila* Idit as a guide to understanding human Irisin and age-related pathologies in brain, heart, and skeletal muscle. In humans and mammals, Irisin is implicated in attenuating various age-associated pathologies in the brain, heart, and skeletal muscle. In *Drosophila*, the Irisin homolog Idit is highly expressed in these three organs and performs important physiological roles in maintaining tissue homeostasis through autophagy control. These results demonstrate the conservation of the Irisin family protein’s role in controlling autophagy and attenuating age-associated pathologies. Protein structures and *Idit* expression pattern results were adapted from Cobb et al., 2023 [46]. Other images were obtained from Wikimedia Commons and are either in the public domain or were created by Laboratories Servier (Cardiovascular_system_-Heart_8--_Smart-Servier.png, Nervous_system_-Brain_10--_Smart-Servier.png, and Tendon_anatomy_1_--_Smart-Servier.png). These images are licensed under CC BY-SA 3.0.

Most notably, autophagy has been shown to be critical for removing toxic protein aggregates, excessive nutrients, and dysfunctional organelles such as damaged mitochondria producing reactive oxygen species—all of which are known accelerators of aging and age-related pathologies. Consistent with Irisin/FNDC5’s protective role against age-associated disorders, including muscle weakening, neurodegeneration, and cardiovascular dysfunction, studies on *Drosophila* Idit revealed critical role of the proteins in skeletal muscle, brain and heart, especially in the context of exercise (Figure 3). Furthermore, Drosophila represents a cost-effective, genetically tractable, and fast genetic model that shares many pathways conserved with humans, including those pivotal for aging, exercise response, neurodegeneration, and cardiac homeostasis. Below, we review in detail how Irisin/FNDC5 may counteract these age-associated pathologies in both mammalian systems and the invertebrate *Drosophila* model.

## 3. FNDC5/Irisin and Musculoskeletal Disorders

### 3.1. FNDC5/Irisin Counteracts Age-Related Sarcopenia in Mammals

Age-associated sarcopenia, defined as the progressive decline in skeletal muscle mass and strength with aging, significantly impairs both quality of life and longevity [61]. Serum Irisin levels correlate positively with measures of muscle strength, such as handgrip strength and short physical performance battery scores, suggesting a role for Irisin in maintaining muscle function [62]. Guo et al. demonstrated that 24-month-old mice with naturally occurring sarcopenia exhibited significant reductions in grip strength, muscle weight, and muscle fiber size compared to young (2-month-old) mice [21]. These changes were accompanied by decreased *FNDC5* mRNA expression in quadriceps femoris, gastrocnemius, and tibialis anterior muscles, as well as reduced serum Irisin levels [21]. Additionally, sarcopenic mice showed the increased expression of atrophic genes (*atrogin-1*, *MuRF-1*, and *Mstn*) and pro-inflammatory cytokines (*IL1β* and *IL6*) [21]. The administration of recombinant Irisin to 14-month-old mice over a four-month period reduced atrophic and inflammatory gene expression while enhancing muscle weight and grip strength, indicating that Irisin can effectively mitigate age-associated sarcopenia [21].

Human clinical studies have also supported the strong link between Irisin and sarcopenia [25]. Several investigations have demonstrated that serum Irisin levels are notably lower in individuals with sarcopenia compared to those without the condition. Additionally, serum Irisin levels have been positively correlated with muscle mass, strength, and function [63,64,65]. These findings collectively highlight Irisin as a promising biomarker and therapeutic target for the prevention and management of age-related sarcopenia, warranting further research to fully understand its role in muscle health and aging.

### 3.2. FNDC5/Irisin Prevents Intervertebral Disc Degeneration

Intervertebral discs, which provide flexibility and cushioning between vertebrae, undergo significant structural and compositional degeneration with aging, leading to conditions such as intervertebral disc degeneration (IVDD) [66]. The dysfunction of the nucleus pulposus (NP), a key cellular component of intervertebral discs, is a hallmark of IVDD [67,68]. Exercise has been shown to mitigate disc degeneration, as evidenced by increased intervertebral disc height and reduced NP apoptosis in mice following a four-week exercise regimen [69]. However, these benefits were significantly attenuated in *FNDC5* knockout mice, highlighting the role of FNDC5 and Irisin in maintaining disc integrity [69].

In vitro studies further support Irisin’s protective effects, showing that it reduces tert-butyl hydroperoxide (TBHP)-induced apoptosis in rat NP cells while promoting autophagy [69]. Both Irisin treatment and *FNDC5* overexpression stimulated autophagy in NP cells, whereas exercise-induced autophagy was absent in *FNDC5* knockout mice [69]. Notably, the Irisin-mediated inhibition of apoptosis was reversed when autophagy was experimentally suppressed, suggesting that autophagy is a key mechanism through which Irisin protects against IVDD [69]. Additionally, Irisin modulates extracellular matrix (ECM) metabolism in NP cells by activating the Hippo signaling pathway, restoring balance disrupted by TNF-α or mechanical injury [70,71]. Irisin also induces anabolic ECM gene expression, including *COL2*, *ACAN*, *TIMP-1*, and *TIMP-3*, further contributing to disc homeostasis [72]. Together, these findings position Irisin as a promising therapeutic target for IVDD.

### 3.3. Irisin’s Complex Role in Bone Homeostasis

Osteoporosis (OP) is often driven by a net reduction in bone mass—a result of an imbalance between bone formation and bone resorption [2]. Irisin has been shown to mitigate osteoporosis caused by ovariectomy; recombinant Irisin injection increased bone strength and mass in mice at a low concentration (100 μg/kg). This protective effect was partly due to the upregulation of osteogenesis-related genes, including *Atf4*, *Runx2*, *Osx*, *Lrp5*, *β-catenin*, *Alp*, and *Col1a1* [73,74].

In a separate model, hind-limb suspension led to elevated sclerostin—a key protein that promotes bone resorption. However, Irisin administration prevented this sclerostin increase, thereby preserving bone mass [17]. Conversely, Irisin can also bind integrin αV (particularly αVβ5), activating the FAK-AKT-CREB pathway and inducing sclerostin, which exacerbates trabecular bone loss in ovariectomized mice [41]. Moreover, Irisin’s effects on bone can be sex-specific; in a low-calcium diet or lactation-induced bone-loss model, male *FNDC5* knockout mice showed greater bone loss than female knockouts, indicating a gender-dependent regulatory mechanism [75].

Taken together, these findings suggest that Irisin plays complex yet influential roles in bone homeostasis, acting through both pro- and anti-sclerostin pathways, as well as upregulating osteogenic genes. Further research is needed to clarify how these mechanisms intersect under different physiological and pathological conditions.

### 3.4. Drosophila FNDC5/Irisin Homolog Mediates Exercise Benefits in Skeletal Muscle

In *Drosophila*, the FNDC5/Irisin homolog, Iditarod (Idit), is highly expressed in muscle tissue and is induced by exercise [60], mirroring regulatory mechanisms seen in mammalian systems. Both chronic physical activity and dPGC-1α upregulation boost *Idit* expression [60]. Notably, muscle-specific *Idit* overexpression enhances endurance, emphasizing that, akin to mammalian Irisin, this protein is central to maintaining and improving muscle performance.

Since *Idit* expression is upregulated by repeated exercise, Idit also appears to be crucial for exercise-induced adaptations. Flies lacking Idit fail to improve their endurance or benefit from exercise training, a stark contrast to wild-type flies that show significant gains under the same protocols [60]. Restoring or overexpressing *Idit* in muscle rescues endurance levels, even in the absence of training [60], paralleling mammalian findings in which Irisin promotes mitochondrial function, boosts energy metabolism, and facilitates autophagy. Moreover, the muscle-specific silencing of *Idit* alone is enough to block exercise benefits, confirming that muscle is the primary site where Idit mediates exercise adaptations.

### 3.5. Drosophila FNDC5/Irisin Homolog Confers Thermotolerance Through Skeletal Muscle

Beyond endurance, Idit also confers thermotolerance, echoing the role of mammalian Irisin in cold adaptation and thermogenesis. Idit-deficient flies display diminished cold survival, whereas *Idit* overexpression counteracts the damaging effects of low temperature [60]. Notably, skeletal muscle-specific *Idit* overexpression similarly confers cold resistance [60]. Given that flies lack brown adipose tissue and Ucp1-dependent thermogenesis, it is plausible that Idit supports thermotolerance through muscle-based mechanisms, potentially including shivering, as described in certain insect species [76].

### 3.6. Drosophila Provides a Tractable Model for Investigating Irisin/FNDC5 Functions

Because *Drosophila* possesses an exoskeleton rather than endoskeletal structures, any potential bone-related effects of Irisin are not observed in the fly model. Nevertheless, the fundamental importance of Irisin in exercise physiology and muscle health is strikingly conserved in *Drosophila*. As sarcopenia and muscle frailty remain pressing issues in aging populations, understanding how Irisin action can be leveraged in both flies and mammals may prove beneficial. In particular, *Drosophila* presents an accessible model for dissecting the molecular underpinnings of Irisin’s role in maintaining muscle strength and homeostasis. Some of these basic principles of Irisin’s biology could also shed light on understanding its role in maintaining bone homeostasis as well.

## 4. FNDC5/Irisin and Neurodegenerative Diseases

### 4.1. Mammalian Blood–Brain Barrier Is Permeable to Secreted Irisins

The blood–brain barrier (BBB) is a highly specialized structure in the central nervous system (CNS) that tightly regulates the passage of molecules between the bloodstream and the brain, providing a protective barrier against potentially harmful substances [77]. Notably, peripheral Irisin has been shown to cross the BBB and elevate the expression of brain-derived neurotrophic factor (*BDNF*) [13], an essential mediator of synaptic plasticity, learning, and memory [78,79]. This Irisin–BDNF interaction is particularly significant in the context of neurodegenerative diseases such as Alzheimer’s disease (AD) and Parkinson’s disease (PD). Multiple studies indicate that exercise-induced Irisin upregulation can help preserve cognitive function by promoting neuronal survival and synaptic integrity through BDNF-dependent pathways [80].

Islam et al. (2021) demonstrated that Irisin plays a critical role in mediating the cognitive benefits of exercise, as the genetic deletion of *Fndc5*/*Irisin* impairs cognitive function in aging and AD models, while peripheral Irisin delivery rescues cognitive deficits and mitigates neuropathology [81]. Furthermore, recent research has expanded our understanding of Irisin’s neuroprotective effects beyond BDNF modulation. Wang et al. (2022) found that Irisin ameliorates neuroinflammation and neuronal apoptosis via the integrin αVβ5/AMPK signaling pathway following intracerebral hemorrhage (ICH), demonstrating its ability to regulate microglial polarization, suppress neutrophil infiltration, and reduce neuroinflammatory damage [82]. These findings underscore the broader neuroprotective role of Irisin in acute brain injuries in addition to chronic neurodegenerative conditions.

Additionally, Wang et al. (2024) provided compelling evidence that Irisin counteracts microglial senescence in tauopathy by enhancing mitochondrial biogenesis through the mitochondrial transcription factor A (TFAM) pathway. This effect was shown to restore mitochondrial oxidative phosphorylation (OXPHOS) and mitigate cognitive decline in a tauopathy mouse model, further implicating Irisin as a potential therapeutic agent for neurodegenerative diseases associated with tau pathology [83].

Together, these studies highlight Irisin’s diverse neuroprotective mechanisms, including the modulation of neurotrophic factors, anti-inflammatory signaling, and mitochondrial metabolism. These findings not only reinforce Irisin’s role in cognitive resilience and neuroprotection but also underscore its therapeutic potential for both acute and chronic neurodegenerative conditions.

### 4.2. Irisin’s Role Against Alzheimer’s Disease

It has been reported that the incidence of Alzheimer’s disease (AD) increases with age [84]. Physical exercise has been shown to promote the degradation of amyloid-β, a key peptide in AD pathogenesis [85]. In humans, cerebrospinal fluid (CSF) Irisin level are positively correlated with BDNF and amyloid beta42 [86]. Notably, Irisin mRNA and protein levels are reduced in the CSF of AD patients and in the hippocampi of mice treated with amyloid-β oligomers (AβOs) [80]. Additionally, FNDC5 knockdown in the mouse brain impairs synaptic plasticity and memory, effects that can be reversed by restoring *FNDC5* expression or administering Irisin. In both human cortical slices and mouse hippocampal slices, Irisin increases cAMP and activates the PKA-CREB pathway. Moreover, the AβO-facilitated nucleus translocation of ATF4 is blocked by Irisin-mediated PKA activation. Finally, swimming exercise significantly alleviated AβO-induced or APP/PS1 ΔE9-induced deficits in synaptic plasticity and memory [80].

In a 3D human neuronal AD model comprising neurons, astrocytes, and oligodendrocytes, Irisin treatment enhanced neprilysin secretion from astrocytes. Neprilysin plays a critical role in degrading amyloid-β (Aβ), a key contributor to AD pathology [87,88,89,90]. Notably, astrocytes in this model exhibited the high expression of integrin β5, a receptor previously identified as an Irisin-binding partner in osteocytes and adipose tissue [41,42,91]. Kim et al. proposed that Irisin interacts with integrin αVβ5 in astrocytes, suppressing ERK-STAT3 signaling and thereby promoting neprilysin secretion [90]. These findings highlight Irisin’s potential to mitigate AD progression by targeting astrocyte-driven Aβ clearance mechanisms.

### 4.3. Irisin’s Role Against Parkinson’s Disease

Parkinson’s disease (PD) is a neurodegenerative disorder that disrupts the nervous system and impairs movement. Similarly to AD, aging is a significant risk factor for PD [92]. The neurotoxin 1-methyl-4-phenyl-1,2,3,6-tetrahydropyridine (MPTP) is known to induce PD-like pathology [93]. Notably, Irisin can mitigate MPTP-induced neuronal apoptosis in the substantia nigra pars compacta and striatum [94].

Moreover, Irisin downregulates apolipoprotein E (ApoE), a protein linked to increased risk for both PD and AD [95,96,97]. Recent evidence also suggests that Irisin may inhibit α-synuclein (α-syn) aggregation, a key pathogenic process in PD [97]. By enhancing the lysosomal degradation of internalized α-synuclein preformed fibrils in neuronal cells, Irisin decreases the seeding of endogenous α-syn misfolding [97].

Zhang et al. further demonstrated that Irisin exerts neuroprotective effects against multiple mitochondrial ROS–generating neurotoxins, including 1-methyl-4-phenylpyridinium (MPP+), MPTP, and rotenone, all of which induce apoptosis [98,99,100,101]. Importantly, both pre- and post-treatment with Irisin attenuated mitochondrial ROS production, mitochondrial dysfunction, and neuronal cell death. Similarly to its activity in osteocytes and adipose tissue, Irisin activates the AKT and ERK signaling pathways via integrin αV receptors, a process blocked by iRGD (an integrin αV antagonist) and by ERK or AKT inhibitors [98]. These findings highlight Irisin’s therapeutic potential in mitigating PD-related neuronal damage through antioxidant and antiapoptotic mechanisms.

### 4.4. The Role of Irisin Homolog in Drosophila Brain

Even though *Drosophila* Idit was not extensively studied in the brain, there is evidence supporting its neuronal function. First, *Idit* expression, estimated using its gene trap transgenic allele, was very high in the central nervous system [60], which was significantly stronger than its expression in muscular tissues. The high expression of Idit in brains suggests its neuronal role. Indeed, the neuron-specific overexpression of *Idit* was able to slightly extend the lifespan of *Drosophila* in adults [60], suggesting that Idit plays a physiological role in the brain in attenuating aging. This observation is consistent with the beneficial function of Irisin in combating neurodegenerative disorders in mice.

It is important to note that, in recent years, the exercise training of *Drosophila* was able to attenuate neurodegenerative pathologies caused by polyglutamine toxicity [102]. Sestrin, which is important for exercise-induced PGC1 upregulation [103,104], was shown to be important in this process [102]; so it is very plausible that Idit is also involved in exercise benefits in attenuating neurodegeneration. Such activities could also be achieved through the upregulation of autophagy, which is critical for attenuating neurodegeneration [105] by removing toxic protein aggregates and dysfunctional reactive oxygen species-producing mitochondria. It is also possible that, in addition to the cell-autonomous role of Idit in controlling autophagy in flies, Irisin has evolved to play an endocrine role in mammals, thus enhancing autophagy, as exemplified above in the therapeutic effects of exogenous Irisin in ameliorating various neurodegeneration-associated pathologies. Therefore, the *Drosophila* model again proves to be highly useful for interrogating biological pathways associated with Irisin-mediated neuroprotection.

## 5. FNDC5/Irisin and Cardiovascular Diseases

### 5.1. Irisin’s Role Against Cardiac Hypertrophy

Cardiac hypertrophy is a hallmark of heart aging, arising from prolonged pressure and volume overload that ultimately leads to heart failure [106,107]. Transverse abdominal aortic constriction (TAC) is a well-established model of pressure overload-induced cardiac hypertrophy [108]. Studies indicate that Irisin mitigates TAC-induced oxidative stress and apoptotic cell death in rats, thereby reducing cardiac hypertrophy [109]. Additionally, some reports show that Irisin downregulates TAC-mediated AKT phosphorylation, a key event driving ROS production and cardiac remodeling [109,110,111].

Evidence also suggests that Irisin-induced autophagy is central to its protective effects on the heart [56,57]. For example, Angiotensin II (AngII) suppresses autophagy flux and induces cardiomyocyte hypertrophy and apoptosis; however, Irisin administration restores autophagy flux and inhibits apoptosis under these conditions [57]. In a related study, Irisin transgenic mice exhibited reduced TAC-induced cardiac hypertrophy, an effect reversed by the pharmacological inhibition of autophagy [56]. Mechanistically, Irisin elevates AMPK and ULK1 phosphorylation in both transgenic mice and neonatal rat ventricular myocytes. The pharmacological inhibition of AMPK or ULK1 eliminates Irisin’s anti-hypertrophic effects, highlighting the importance of this signaling axis [56].

Notably, TAC- or AngII-induced cardiac hypertrophy elevates *FNDC5* expression, resulting in increased Irisin protein [10]. Once elevated, Irisin activates AMPK and inhibits mTOR, without significantly impacting MAPKs such as JNK, ERK, or p38 [10]. The inhibition of AMPK using compound C blocks Irisin’s cardioprotective effect, underscoring AMPK’s pivotal role in Irisin-mediated cardiac hypertrophy attenuation [10]. As AMPK activation, mTOR inhibition, and ULK1 activation are interconnected in promoting autophagy, Irisin’s regulation of this signaling pathway is crucial for mitigating various cardiac pathologies, as discussed below.

### 5.2. Irisin’s Role Against Myocardial Infarction

Myocardial infarction (MI) remains a leading cause of morbidity and mortality worldwide due to irreversible myocardial cell death. Optic atrophy 1 (OPA1) is crucial for mitochondrial fusion and fate determination via mitophagy [112,113,114], and emerging evidence implicates OPA1 in myocardial ischemic injury [115,116]. Xin et al. observed that *OPA1* expression was suppressed in infarcted mouse hearts and under hypoxic conditions [58]. Hypoxia also reduced key mitophagy proteins (Parkin, LC3II, p62, and ATG5), leading to cardiomyocyte apoptosis, even as the mitochondrial membrane potential was disrupted [58].

However, Irisin supplementation restored these mitophagy-associated proteins, highlighting its role in OPA1-dependent mitophagy and cardiomyocyte protection under hypoxia [58]. Beyond this mechanism, Irisin enhances cardiac repair by improving the function of cardiac progenitor cells (CPCs). The transplantation of Irisin-pretreated CPCs into MI sites significantly improved cardiac function, in part through enhanced cardiomyocyte differentiation, proliferation, angiogenesis, and reduced fibrosis and apoptosis [117]. Similarly, Q. Liao et al. demonstrated that Irisin promotes ERK-dependent angiogenesis, thereby further aiding MI recovery [118].

In addition, histone deacetylases (HDAC) play a critical role in ischemic heart injury [119,120]. The overexpression of *HDAC4* exacerbates hypoxia/reoxygenation (HR)-induced apoptosis in cardiomyoblasts, but Irisin alleviates this damage by increasing HDAC4’s interaction with SUMO-1, ultimately targeting HDAC4 for degradation [121]. Collectively, these findings underscore Irisin’s multifaceted cardioprotective effects following MI.

### 5.3. Irisin’s Role Against Cardiovascular Aging

Chronic inflammation is a major risk factor for cardiovascular diseases, including atherosclerosis [122]. Irisin downregulates pro-inflammatory genes—such as *IL-6*, *VCAM-1*, *ICAM-1*, and *MCP-1*—by suppressing NF-κB activity, thereby reducing apoptotic cell death and slowing atherosclerosis progression [123]. In diabetic *apoE*^−/−^ mice, Irisin administration activates the AMPK–PI3K–AKT–eNOS signaling pathway, elevating serum NO levels and consequently decreasing inflammatory cytokines (*IL-6*, *TNF-α*, *ICAM-1*, *VCAM-1*, and *MCP-1*) [124]. This anti-inflammatory effect lessens apoptosis in aortas, which is otherwise aggravated by diabetes [124]. Consistent with these findings, K. Inoue et al. reported that exercise not only increases AMPK, AKT, and eNOS activity but also raises circulating Irisin and NO levels, resulting in reduced arterial stiffness [125]. Irisin has also been shown to lower blood pressure by stimulating NO release [126].

Moreover, exercise-derived extracellular vesicles (EVs) containing Irisin can induce *Dnajb3/Hsp40* expression in blood vessels, which in turn stabilizes SIRT6, ultimately delaying vascular aging [45]. Irisin similarly promotes autophagy in vascular smooth muscle cells (VSMCs), protecting against vascular calcification by curbing pyroptosis through reduced ROS levels [127]. In cardiac microvascular endothelial cells (CMECs), Irisin alleviates oxidative stress caused by high glucose and lipids by activating ERK1/2 [128]. Activated ERK1/2 induces *SOD-1/2* and *HO-1* expression via Nrf2 translocation, thereby preventing CMEC apoptosis [128]. Additionally, Irisin diminishes oxLDL-induced ROS by activating the AKT/mTOR/Nrf2 pathway, further lowering inflammation and vascular injury [129]. Collectively, these studies highlight Irisin’s broad anti-aging effects on the cardiovascular system.

### 5.4. The Role of Irisin Homolog in Drosophila Heart

In addition to brain and skeletal muscle, Idit was also highly expressed in heart tissue [60]. Therefore, the role of Idit in the heart is crucial for mediating exercise benefits. Previously, pacing stress-induced cardiac failure, which is aggravated by aging, was shown to be suppressed by chronic exercise training [130,131]. Interestingly, Idit deficiency completely nullified these protective effects and made the heart remarkably sensitive to pacing stress [60]. Conversely, the overexpression of *Idit* mimicked exercise benefits even in the absence of actual training; both trained and untrained flies with elevated *Idit* expression were protected from pacing stress-induced cardiac failure [60].

Importantly, autophagy was also critically regulated by Idit in cardiac tissue. Although autophagosomes, monitored through Atg8 puncta, were strongly upregulated after exercise in wild-type flies, flies lacking Idit exhibited a reduced exercise response during autophagy, suggesting that Idit is necessary for the robust autophagy response typically associated with endurance training [60]. The restoration or overexpression of *Idit* in the heart rescued autophagy, which was consistent with elevated resilience against cardiac stress, underscoring that autophagy induction is part of the cardioprotective mechanism conferred by Idit. Mitochondria are often associated with Atg8 in normal exercised hearts, but this association was not observed in Idit-deficient hearts [60], suggesting that Idit is necessary for cardiac mitophagy. The requirement of Idit in cardiac autophagy is also consistent with the role of Idit in mediating Atg1/Atg13-dependent autophagic flux in fly eyes and mammalian cells [60]. Again, the role of Idit is tissue-autonomous, so its mechanism is mainly mediated through either autocrine or transmembrane functions. But such regulation seems to have evolved to involve endocrine functions in mammalian organisms, as reviewed above.

## 6. FNDC5/Irisin and Diabetes

Diabetes incidence in older adults is rising rapidly [132], with over 25% of individuals aged 65 and older affected [133]. Irisin levels have been shown to be inversely correlated with both type 1 and type 2 diabetes [134,135,136]. Because mitochondrial function is closely tied to insulin sensitivity, its impairment often leads to insulin resistance (IR) [137]. Irisin has been reported to induce the “browning” of white adipose tissue (WAT), transforming it into a brown adipose tissue (BAT)-like phenotype. This BAT-like tissue contains higher mitochondrial density and can thereby improve insulin resistance [29]. A key mediator of this browning process is uncoupling protein 1 (UCP1), whose upregulation in WAT promotes thermogenesis and enhances energy expenditure [29]. Consistent with this, Irisin has also been shown to attenuate IR by increasing mitochondrial content in C2C12 myoblasts [54].

Glucose transporter 4 (GLUT4) is highly expressed in adipocytes and myocytes, where it plays a critical role in facilitating glucose uptake into cells [138]. Reduced GLUT4 expression or impaired translocation to the plasma membrane are key drivers of insulin resistance (IR). Irisin administration has been shown to activate AMPK, thereby increasing GLUT4 expression in human skeletal muscle cells [139]. Additionally, Irisin may enhance glucose uptake by promoting AMPK-dependent GLUT4 translocation to the plasma membrane, as observed in type 2 diabetes models and L6 myoblasts [140,141].

Beyond GLUT4 regulation, Irisin has protective effects on pancreatic β-cells, which are vulnerable to free fatty acid (FFA)-induced inflammation. In a high-fat diet (HFD) model, Irisin improved glucose tolerance by reducing inflammation in pancreatic islets [142]. This anti-inflammatory effect may involve the modulation of FOXO1, an upstream transcription factor, through the PI3K-AKT pathway [142]. Collectively, these findings suggest that Irisin improves systemic glucose homeostasis by enhancing GLUT4 activity and protecting β-cell function.

Recent studies have further highlighted Irisin’s role in metabolic regulation. For example, Irisin has been shown to enhance mitochondrial biogenesis and function in metabolic tissues via the activation of the AMPK-PGC-1α pathway [143]. Additionally, Irisin exhibits anti-inflammatory properties by modulating the TLR4/MyD88 signaling pathway, leading to a reduction in pro-inflammatory cytokine production [144]. Furthermore, the Irisin-stimulated browning of white adipocytes can also contribute to improved systemic insulin sensitivity [145].

Although the role of Idit in regulating hemolymph trehalose (the functional equivalent of blood sugar in *Drosophila*) has not been directly examined, we can speculate that it may play a systemic role in carbohydrate homeostasis through modulating neuromuscular metabolism and exercise response. Trehalose is known to be critical for exercise performance [146], and other exercise mediators, such as Sestrins [147], can influence hemolymph trehalose levels [148]. Thus, it is plausible that Idit, as a key exercise-responsive factor, similarly contributes to maintaining optimal trehalose balance and energy availability in *Drosophila*.

## 7. Irisin’s Role in Age-Related Diseases Across Various Organs

Recent investigations revealed Irisin’s role in mitigating age-related pathologies in other organ systems, beyond the cardiovascular and neuromuscular systems described above. In the liver, Irisin has been shown to regulate inflammatory processes and lipid metabolism, helping to alleviate inflammation-mediated metabolic syndrome and reduce the risk of age-related liver conditions such as hepatosteatosis [24,26]. In addition to liver, Irisin plays an important role in the other gastrointestinal organs, such as pancreas and intestines, by modulating inflammation and oxidative stress, thus potentially mitigating diseases such as pancreatitis, inflammatory bowel disease, and pancreatic and intestinal carcinogenesis [26]. In the kidney, Irisin demonstrates protective effects against renal fibrosis and oxidative stress, which contribute to chronic kidney disease (CKD), which is associated with aging [44]. In the lungs, Irisin has been linked to reduced oxidative stress and inflammation, providing protection against conditions such as chronic obstructive pulmonary disease (COPD) and acute lung injury—common in aged individuals [23]. Additionally, Irisin may have an anti-aging role in the skin, as it has been observed to promote dermal health by reducing inflammation and supporting tissue repair [149]. Collectively, these findings underscore Irisin’s potential as a therapeutic target for a wide range of age-associated diseases across various organs, emphasizing its systemic relevance in the aging process.

## 8. Conclusions

Improvements in public health have greatly increased life expectancy, but aging still brings challenges such as physical decline and age-related diseases. With the global aging population growing steadily, these conditions are becoming pressing social and healthcare challenges. Preventing and treating age-related diseases is, therefore, critically important. Unfortunately, the mechanisms of aging remain incompletely understood, making it difficult to develop targeted therapeutic strategies. Exercise has emerged as a promising intervention for combating age-associated diseases, but the underlying mechanisms remain unclear. Irisin, an exercise-induced myokine, has been shown to counteract several age-related conditions. Interestingly, Irisin levels decrease significantly with age, underscoring the importance of understanding its mechanisms in mitigating age-related diseases.

In this review, we explored how Irisin contributes to the amelioration of age-associated diseases (Figure 1) as an exercise-inducible protein (Figure 2), and examined insights from studies on *Drosophila* Idit, an evolutionarily conserved homolog of Irisin (Figure 3). Evidence from *Drosophila* highlights that, beyond its well-studied endocrine functions, the Irisin/FNDC5 family also has critical cell-autonomous roles in regulating autophagy. Autophagy plays a pivotal role in maintaining neuromuscular and cardiac tissue integrity, which are often compromised with age. However, the precise mechanisms by which membrane-bound and secreted Irisin/FNDC5 family proteins enhance autophagy and counteract age-associated pathologies remain to be fully elucidated.

Future studies aimed at understanding the molecular mechanisms through which Irisin/FNDC5/Idit regulates autophagy could offer new strategies for addressing age-related disorders across various organ systems. While the *Drosophila* model provides valuable insights, translating these findings into human biology will require further validation using mammalian models such as human tissue cultures and mouse experiments. Unraveling these pathways may pave the way for innovative therapeutic approaches, providing hope for combating the significant health challenges faced by our aging society.

## Abbreviations

The following abbreviations are used in this manuscript:

AD	Alzheimer’s Disease
AKT	Protein Kinase B
AMPK	AMP-Activated Protein Kinase
APP	Amyloid Precursor Protein
Atg	Autophagy-Related Gene
BAT	Brown Adipose Tissue
BBB	Blood–Brain Barrier
BDNF	Brain-Derived Neurotrophic Factor
CMEC	Cardiac Microvascular Endothelial Cell
CNS	Central Nervous System
CPC	Cardiac Progenitor Cell
CSF	Cerebrospinal Fluid
ECM	Extracellular Matrix
ERRα	Estrogen-Related Receptor Alpha
EV	Extracellular Vesicle
FFA	Free Fatty Acid
FNDC5	Fibronectin Type III Domain-Containing Protein 5
FOXO1	Forkhead Box Protein O1
GLUT4	Glucose Transporter Type 4
HFD	High-Fat Diet
HR	Hypoxia/Reoxygenation
ICAM-1	Intercellular Adhesion Molecule 1
IL-1β	Interleukin 1 Beta
IL-6	Interleukin 6
IR	Insulin Resistance
IVDD	Intervertebral Disc Degeneration
JNK	c-Jun N-terminal Kinase
LC3II	Microtubule-Associated Protein 1A/1B-Light Chain 3II
MCP-1	Monocyte Chemoattractant Protein-1
mTOR	Mechanistic Target of Rapamycin
NP	Nucleus Pulposus
NF-κB	Nuclear Factor Kappa B
NO	Nitric Oxide
OPA1	Optic Atrophy 1
PGC-1α	Peroxisome Proliferator-Activated Receptor Gamma Coactivator-1 Alpha
PI3K	Phosphoinositide 3-Kinase
ROS	Reactive Oxygen Species
SIRT1	Sirtuin 1
SOD2	Superoxide Dismutase 2
SUMO-1	Small Ubiquitin-Like Modifier 1
TAC	Transverse Abdominal Aortic Constriction
TFEB	Transcription Factor EB
TGF-β	Transforming Growth Factor Beta
TIMP	Tissue Inhibitor of Metalloproteinase
TNF-α	Tumor Necrosis Factor Alpha
ULK1	Unc-51-Like Autaophagy Activating Kinase 1
VCAM-1	Vascular Cell Adhesion Molecule 1
VSMC	Vascular Smooth Muscle Cell
WAT	White Adipose Tissue
